# Bone Mineral Density in Antiretroviral Therapy‐Naïve HIV‐1–Infected Young Adult ‐Women Using Depot Medroxyprogesterone Acetate or Nonhormonal Contraceptives in Uganda

**DOI:** 10.1002/jbm4.10446

**Published:** 2020-12-21

**Authors:** Flavia Kiweewa Matovu, Martin Nabwana, Noah Kiwanuka, Delia Scholes, Esther Isingel, Monica L Nolan, Mary G Fowler, Philippa Musoke, John M Pettifor, Todd T Brown, Mags E Beksinska, for the BONE CARE Study Team

**Affiliations:** ^1^ Makerere University‐Johns Hopkins University (MU‐JHU) Research Collaboration Kampala Uganda; ^2^ Makerere University College of Health Sciences Kampala Uganda; ^3^ Kaiser Permanente Washington Health Research Institute Seattle WA USA; ^4^ Johns Hopkins University School of Medicine Baltimore MD USA; ^5^ SAMRC/Wits Developmental Pathways for Health Research Unit, Faculty of Health Sciences University of the Witwatersrand Johannesburg South Africa; ^6^ Maternal Adolescent & Child Health Research Unit, Faculty of Health Sciences University of the Witwatersrand Johannesburg South Africa

**Keywords:** ANTIRETROVIRAL THERAPY‐NAÏVE, BONE MINERAL DENSITY, DEPOT MEDROXYPROGESTERONE ACETATE, HUMAN IMMUNODEFICIENCY VIRUS

## Abstract

Most studies evaluating BMD in human immunodeficiency virus (HIV)‐infected populations have focused on antiretroviral therapy (ART)‐experienced patients. In this study, the association between HIV‐1 and/or depot medroxyprogesterone acetate (DMPA) and BMD among untreated HIV‐1–infected women in a resource‐limited setting was assessed before long‐term exposure to ART. The data were then compared with that of the 2005–2008 United States National Health and Nutrition Examination Survey data for non‐Hispanic White and Black women. Women aged 18–35 years, recruited from health facilities in Kampala, Uganda, were classified based on their combination of HIV‐1 status and DMPA use: (i) HIV‐1–infected current DMPA users, (ii) HIV‐1–infected previous DMPA users, (iii) HIV‐1–infected nonhormonal‐contraceptive users, and (iv) HIV‐uninfected nonhormonal‐contraceptive users. All HIV‐1–infected women reported being ART‐naïve at baseline. BMD was measured at the lumbar spine, total hip, and femoral neck using DXA. Multivariate linear regression was used to assess the association between HIV‐1 and/or DMPA and BMD *Z*‐scores. Baseline data were analyzed for 452 HIV‐1–infected (220 nonhormonal users, and 177 current and 55 previous DMPA users) and 69 HIV‐1–uninfected nonhormonal‐contraceptive users. The mean age was 26.1 years (SD, 4.2) with a median duration of DMPA use among current users of 24.0 months [medians (interquartile range), 12‐48]. A higher proportion of HIV‐1–infected previous (12.7%) or current DMPA users (20.3%) and nonhormonal users (15.0%) had low BMD (*Z*‐score ≤−2 at any of the three sites) compared with age‐matched HIV‐1–uninfected women (2.9%). HIV‐1 infection and DMPA use were independently associated with significantly lower mean BMD *Z*‐scores at all sites, with the greatest difference being among HIV‐1–infected current DMPA users (5.6%–8.0%) versus uninfected nonhormonal users. Compared with non‐Hispanic White and Black women, the Ugandan local reference population had generally lower mean BMD at all sites. Newer treatment interventions are needed to mitigate BMD loss in HIV‐1–infected women in resource‐limited settings. © 2020 The Authors. *JBMR Plus* published by Wiley Periodicals LLC. on behalf of American Society for Bone and Mineral Research.

## Introduction

In sub‐Saharan Africa, the human immunodeficiency virus (HIV‐1)‐burden remains high, particularly among women,^(^ ^1^ ^)^ and many more individuals are living longer with HIV‐1 because of expanded access to antiretroviral therapy (ART) coupled with rising longevity.^(^ ^2^, ^3^ ^)^ A number of studies have demonstrated accelerated BMD loss, higher rates of osteopenia and osteoporosis,^(^ ^4^ ^)^ and subsequent fractures^(^ ^5^, ^6^, ^7^, ^8^ ^)^ over time among HIV‐1–infected individuals compared with the general population.

Though underlying mechanisms leading to accelerated bone loss in HIV‐1–infected persons are still unclear, they are believed to be multifactorial and include both traditional and HIV‐specific risk factors.^(^ ^9^, ^10^, ^11^ ^)^ Based on physiological, psychological, and lifestyle factors, HIV‐1–infected persons are likely to have many of the traditional risk factors for low BMD such as physical inactivity, low body weight, nutritional deficiencies (including inadequate calcium and vitamin D intake), depression, smoking, heavy alcohol use, and oligo/amenorrhea.^(^ ^12^, ^13^, ^14^, ^15^ ^)^ Among the nontraditional causes, a direct effect of HIV‐1 and its treatment have been most often cited.^(^ ^16^, ^17^ ^)^ It is not yet known how much of the BMD loss among HIV‐1‐infected patients is caused by the HIV‐1 infection per se or how much can be attributed to the consequences of ART.

To date, most studies evaluating BMD in HIV‐1‐infected populations have focused on ART‐experienced patients. Furthermore, most other studies examining bone mass in HIV‐1‐infected persons that we are aware of have had an overwhelming majority of men (generally >85%), limiting the generalizability of the findings to women. There are limited data on bone mass in HIV‐1‐infected untreated adult women in resource‐limited settings. Consequently, there is a need to quantify the prevalence of low BMD and identify associated risk factors for low BMD in untreated HIV‐1‐infected women before long‐term exposure to ART with the objective of optimizing their bone health.

Among women of reproductive age, the choice of contraception also impacts their BMD. Of greatest concern is the concurrent use of depot medroxyprogesterone acetate (DMPA; Depo Provera) among young HIV‐1‐infected women, as DMPA use also leads to accelerated BMD loss and increased later risk for fractures.^(^ ^18^, ^19^, ^20^) However, recovery of BMD has been documented to occur after discontinuation.^(^ ^21^, ^22^ ^)^

The aim of this study was to therefore assess the association of DMPA use with low BMD among the ART‐naïve HIV‐1–infected women enrolled in the BONE: CARE (BONE: Contraception and Anti‐Retroviral Effects) study at the Makerere University–Johns Hopkins University (MU‐JHU) Research Collaboration in Kampala, Uganda. In addition, we report data on the prevalence of low BMD among ART‐naïve HIV‐1–infected women compared with their uninfected counterparts. We further compare our data to that of the 2005–2008 United States National Health and Nutrition Examination Survey (NHANES) data for non‐Hispanic White and Black women.^(^ ^23^ ^)^

## Participants and Methods

### Study population

Women were sequentially recruited from 11 HIV‐care and general‐health facilities in and around Kampala, Uganda, and classified based on their combination of HIV‐1 status and DMPA use: (i) HIV‐1–infected current DMPA users, (ii) HIV‐1–infected previous DMPA users, (iii) HIV‐1–infected nonhormonal‐contraceptive users, and (iv) HIV‐1–uninfected nonhormonal‐contraceptive users. Study entry criteria included age between 18 and 35 years and documented HIV‐1–infection status. Current DMPA users had been using DMPA for contraception at enrollment for at least six consecutive months, whereas previous DMPA users had not used DMPA for at least 6 months prior to enrollment but had ever used it in the past regardless of the duration of use. Nonhormonal groups (infected and uninfected) consisted of current users of the TCu380A copper intrauterine device or condoms and had not used DMPA or any other hormonal method for more than three consecutive months in the last 2 years. All HIV‐1–uninfected women had to be using nonhormonal contraception.

Major exclusion criteria included low CD4 cell count below 100 cells/μl, use of ART for over 10 days prior to enrollment, pregnancy, or breastfeeding history in the last 6 months prior to enrollment, intention to become pregnant for the 2‐year study duration, and taking medications or having a pre‐existing medical condition known to affect bone metabolism. HIV‐1–uninfected women were excluded if they had used oral pre‐exposure or postexposure prophylaxis in the last 6 months.

The study protocol was approved by the Uganda Virus Research Institute Ethics Committee (GC/127/16/09/524), the Uganda National Council for Science and Technology (HS 1942), and the Human Research Ethics Committee at the University of the Witwatersrand, South Africa (M150858). Written informed consent for study participation was obtained from all participants.

### Study procedures

#### *Clinical assessments*

Using structured pretested questionnaires, we collected information on demographics, medical (including concomitant medications), reproductive, and dietary history. Physical activity was assessed using the International Physical Activity Questionnaire.^(^ ^24^ ^)^ Physical activity was categorized per World Health Organization physical activity guidelines, which recommend ≥150 min of moderate‐intensity physical activity or ≥75 min of vigorous‐intensity physical activity in a week for adults aged 18 to 64 years.^(^ ^25^ ^)^ Data on CD4 cell counts and viral load were abstracted from the participants' primary health facility records.

#### *BMD assessments*

BMD of the lumbar spine (LS; L1‐L4), total hip (TH), and femoral neck (FN) were measured using DXA according to the International Society for Clinical Densitometry guidelines.^(^ ^26^ ^)^ Scans were performed using the Hologic Explorer (Hologic, Bedford, MA, USA) interfaced with Apex system software (version 2.3.2). BMD *Z*‐scores of HIV‐infected women were calculated by comparing their values with the mean and SD of the gender‐, age‐, and race‐matched Ugandan control population. Low BMD was defined as a *Z*‐score ≤−2.0 SD at any of the three sites (LS, TH, and FN) in accordance with the National Osteoporosis Foundation guidelines definition.^(^ ^27^, ^28^ ^)^

#### *Laboratory methods*

A urine human chorionic gonadotropin test was performed for each participant prior to a DXA scan. Viral‐load testing was done using the COBAS AmpliPrep/COBAS TaqMan Assay (version 2.0; Roche Molecular Systems, Pleasanton, CA, USA). The reportable range for the TaqMan assay is 20–10,000,000 copies/ml. Testing was performed at the College of American Pathologists‐certified Infectious Diseases Institute Core Research Laboratory in Kampala, Uganda.

#### *Quality control*

The DXA equipment was standardized daily using a phantom as prescribed in the manufacturer's instructions. A DXA expert and bone specialist (JMP) guided the planning and execution of the DXA investigations. A central reading of 10% of DXAs was provided as part of quality assurance. Any scans requiring correction were sent back electronically to replace the original scan results (<2%). JMP and the DXA technicians were blinded to the participant's ART and contraception.

### Statistical analysis

Descriptive analyses were done using means (SD) and medians (interquartile range [IQR]) for continuous variables and percentages for categorical variables. Baseline characteristics were compared among the four groups using Pearson's chi‐squared test for categorical analysis or Fisher's exact test and one‐way analysis of variance for continuous variables. We compared our data with that of the non‐Hispanic White and Black reference data in the 2005–2008 US NHANES.^(^ ^23^ ^)^

The primary outcome was mean baseline‐BMD *Z*‐scores. We compared baseline BMD *Z*‐scores among all treatment‐naive HIV‐1‐infected (nonhormonal, previous and current DMPA users) to that of HIV‐1–infected nonhormonal users. We further compared BMD *Z*‐scores among previous and current DMPA users with that of HIV‐1–infected nonhormonal users, as well as the HIV‐1–uninfected comparison group. Differences in mean BMD *Z*‐scores between the aforementioned groups were assessed using Student *t* tests. Multivariable linear regression was used to estimate adjusted differences between the two groups. Demographic variables with a crude (unadjusted) *p* ≤ 0.2 at univariate modeling and those that are known confounders a priori were selected for inclusion in the multivariable model. Statistically significant differences in levels of mean BMD *Z*‐scores were tested at a *p* ≤ 0.05. We further computed percent differences in mean BMD of HIV‐1–infected nonhormonal and previous and current DMPA users versus uninfected controls.

Analyses were done using Stata software (release 15; Stata Corp, College Station, TX, USA).

## Results

### Sociodemographic and clinical characteristics

Between March 2015 and October 2017, we screened 549 women. Of these, 15 were excluded because of pregnancy and 13 were excluded for other reasons (unstable location, unexplained amenorrhea for over 8 years, declined study participation, or use of combined oral‐contraceptive pills) resulting in a final analytical sample of 521 women: 452 HIV‐1–infected and 69 HIV‐1–uninfected women. The median time between ART initiation and enrollment was 0 days (IQR, 0‐2 days).

**Fig 1:**
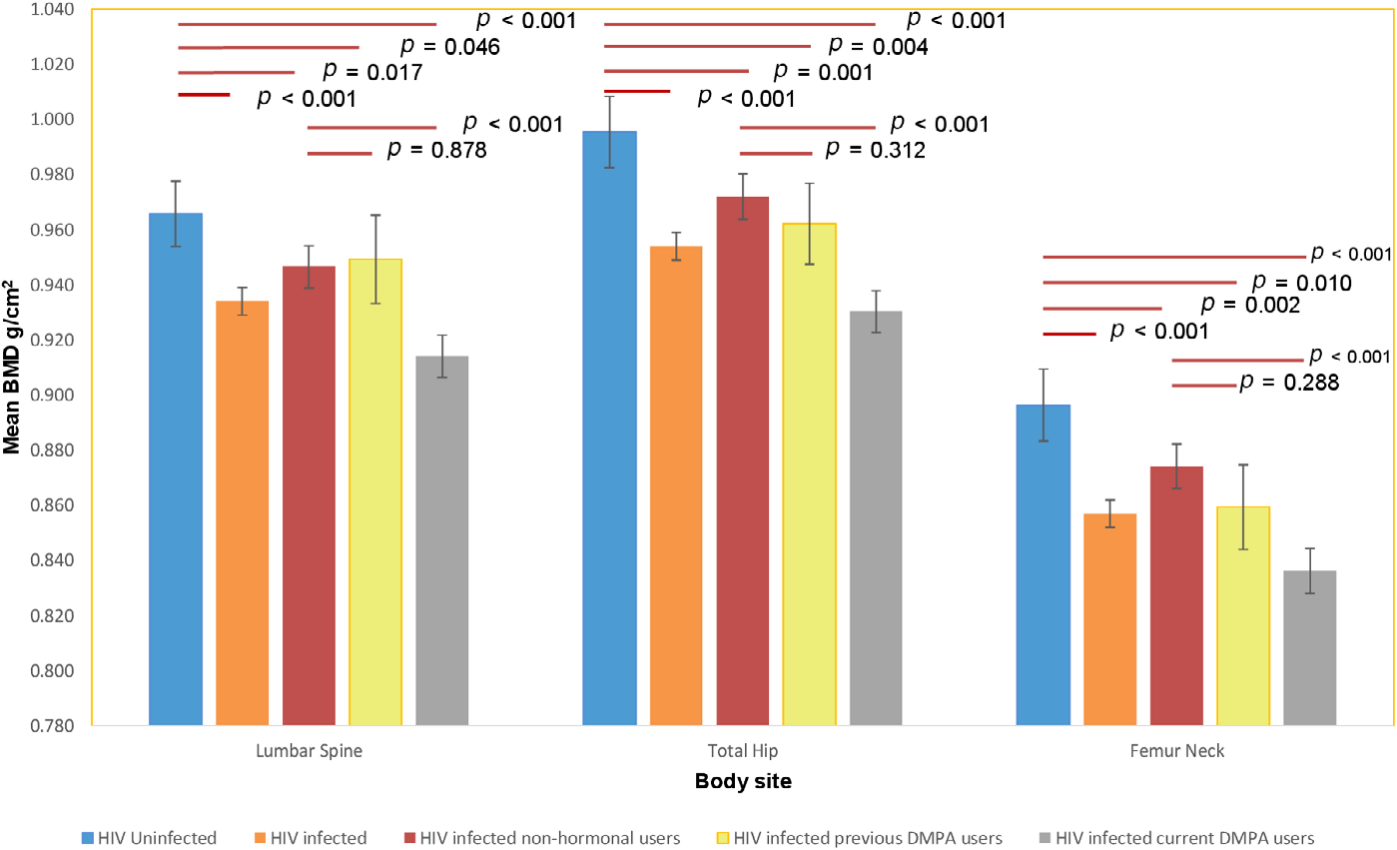
Mean BMD at the different body sites by human immunodeficiency virus (HIV)‐1 infection and contraception status.

Baseline characteristics are given in Table 1. The mean age was 26.1 years (SD, 4.2 years), 227 (43.6%) were married or cohabiting, 436 (83.7%) had ever been pregnant with a median parity of 2 (IQR, 1‐3 parity), and 313 (60.1%) had a history of DMPA use. Of the 313 women who had ever used DMPA, 176 (56.2%) were current users with a median duration of use of 24 months (IQR, 12‐48 months). The majority of participants (75.2%) reported a history of breastfeeding, with median cumulative breastfeeding duration of 26 months (IQR, 15‐43 months). Compared with HIV‐1–uninfected women, HIV‐1–infected women were more likely to have less education, higher parity, and to have ever breastfed: *p* < 0.001 for all the variables. The median CD4 cell count among the HIV‐1–infected groups was 670 cells/μl (IQR, 411‐859 cells/μl). HIV‐1–infected women had significantly lower mean BMD compared with the HIV‐1–uninfected age‐matched comparison group at all sites, with the current DMPA users having the lowest means (*p* < 0.05 for all; Fig. 1). However, there were no differences in BMD between younger (18–24 years) and older women^(^ ^25^, ^26^, ^27^, ^28^, ^29^, ^30^, ^31^, ^32^, ^33^, ^34^, ^35^ ^)^ in the different study groups (data not shown). Similarly, there was no difference in mean BMI across the different groups.

**Table 1:** Baseline Characteristics of Women in the BONE: CARE Study by HIV‐1 Status and Contraceptive Method

Characteristic^a^	Total (*N* = 521)	HIV‐1– infected current DMPA users (*n* = 177)	HIV‐1– infected previous DMPA users (*N* = 55)	HIV‐1–infected nonhormonal users (*n* = 220)	HIV‐1– uninfected nonhormonal users (*n* = 69)	*p* Value ^b^
Age (y)	26.1 (4.2)	26.5 (3.8)	27.5 (4.0)	26.0 (4.4)	24.4 (4.0)	<0.001
18–24	202 (38.8)	62 (35.0)	13 (23.6)	93 (42.3)	34 (49.3)	
25–35	319 (61.2)	115 (65.0)	42 (76.4)	127 (57.7)	35 (50.7)	0.013
Education						
None	30 (5.8)	17 (9.6)	1 (1.8)	12 (5.5)	0(0)	
Primary	198 (38)	81 (45.8)	31 (56.4)	74 (33.6)	12 (17.4)	
Secondary	250 (48)	75 (42.4)	19 (34.5)	117 (53.2)	39 (56.5)	
Tertiary	43 (8.3)	4 (2.2)	4 (7.3)	17 (7.7)	18 (26.1)	<0.001
Marital status						
Single	112 (21.5)	26 (14.7)	10 (18.2)	59 (25.8)	17 (24.6)	
Married/cohabiting	227 (43.6)	99 (55.9)	24 (42.6)	81 (36.8)	23 (33.3)	
Causal partner	126 (24.2)	36 (20.3)	10 (18.2)	53 (24.1)	27 (39.1)	
Divorce/separated/widow	56 (10.7)	16 (9.1)	11 (20.0)	27 (12.3)	2 (2.9)	<0.001
Earns income	362 (69.5)	137 (77.4)	37 (67.3)	154 (70.0)	34 (49.3)	<0.001
Median monthly income (USD)	40.7 (27.1, 70.6)	40.7 (27.1, 65.1)	27.1 (19.0, 48.9)	40.7 (27.1, 81.4)	54.3 (40.7, 81.4)	0.020
Ever‐used DMPA	315 (60.5)	177 (100)	55 (100)	69 (31.4)	14 (20.3)	<0.001
Median duration of DMPA use (mo)	N/A	24 (12, 48)	N/A	N/A	N/A	
Age at menarche (y)	14.2 (1.6)	14.1 (1.6)	14.3 (1.7)	14.3 (1.6)	14.0 (1.4)	0.352
Ever pregnant	436 (83.7)	171 (96.6)	52 (94.5)	180 (81.8)	33 (47.8)	<0.001
Parity	2.2 (1.6)	2.6 (1.5)	3.0 (1.8)	2.0 (1.5)	1.0 (1.4)	<0.001
Ever breastfed	390 (74.9)	163 (92.1)	49 (89.1)	153 (69.5)	25 (36.2)	<0.001
Median duration of breast feeding	26 (15, 43)	27 (15, 42)	30 (18, 48)	24 (15, 42)	28 (18, 50)	0.760
Currently drinks alcohol	142 (27.3)	66 (37.3)	18 (32.7)	50 (22.7)	8 (11.8)	<0.001
No. of beers	4.8 (10.2)	3.7 (5.6)	5.2 (7.4)	4.4 (5.3)	16.1 (36.0)	0.975
Current smoker^c^	12 (54.5)	6 (66.7)	0 (0.0)	6 (54.5)	0 (0.0)	0.447
Physical activity (min/wk)^d^						
Vigorous (≥75)	55 (77.5)	17 (73.9)	11 (100)	18 (72)	9 (75)	0.245
Moderate (≥150)	169 (62.8)	63 (67.0)	20 (83.3)	58 (53.2)	28 (66.7)	0.027
Median CD4 cell count (cells/μl)	670 (411, 859)	745 (484, 927)	709 (480, 842)	560 (362, 795)	N/A	0.001
BMI (kg/m^2^)	24.7 (4.4)	25.6 (4.6)	24.7 (4.1)	24.3 (4.2)	24.0 (4.1)	0.012
Median viral load (log^10^ copies/mL)	4.1 (3.2, 4.6)	4.1 (3.1, 4.5)	3.9 (3.6, 4.6)	4.1 (3.1, 4.6)	N/A	0.393
Mean BMD (g/cm^2^)						
Lumbar spine	0.938 (0.110)	0.914 (0.102)	0.949 (0.119)	0.947 (0.114)	0.966 (0.098)	0.002
Total hip	0.960 (0.115)	0.931 (0.101)	0.965 (0.108)	0.970 (0.124)	0.995 (0.107)	<0.001
Femoral neck	0.863 (0.116)	0.837 (0.108)	0.861 (0.114)	0.873 (0.120)	0.896 (0.108)	0.001
Low BMD *Z*‐score (≤−2)						
Lumbar spine	−0.383 (1.131)	−0.630 (1.070)	−0.353 (1.212)	−0.313 (1.162)	0.000 (0.985)	
Total hip	−0.452 (1.120)	−0.734 (1.012)	−0.524 (1.033)	−0.349 (1.205)	0.000 (0.985)	<0.001
Femoral neck	−0.409 (1.091)	−0.654 (1.034)	−0.519 (1.053)	−0.312 (1.131)	0.000 (0.985)	<0.001
Low BMD *Z*‐score (≤−2)						
Lumbar spine	43 (8.2)	20 (11.3)	4 (7.3)	18 (8.2)	20 (11.3)	0.068
Total hip	45 (8.6)	22 (12.4)	2 (3.6)	21 (9.5)	0 (0.0)	0.002
Femoral neck	33 (6.3)	17 (9.6)	2 (3.6)	13 (5.9)	1 (1.4)	0.092

^a^ Mean or proportions unless stated otherwise.

^b^ Difference in baseline characteristic across groups.

^c^ Among those who have ever smoked.

^d^ Vigorous physical activity over a shorter duration (at least 75 min) has the same health benefits as moderate physical activity performed over a longer period of time (at least 150 min). Some women contributed to both categories of physical activity.

BONE: CARE = BONE: Contraception and Anti‐Retroviral Effects; DMPA = depot medroxyprogesterone acetate; HIV‐1 = human immunodeficiency virus.

### BMD *Z*‐scores in untreated HIV‐1–infected versus HIV‐1–uninfected women

Low BMD (*Z*‐score ≤−2) at any of the three sites was found in 16.8% (76 of 452) of HIV‐1–infected women versus 2.9% (2 of 69) of the HIV‐1–uninfected comparison group (Table 2). Mean BMD *Z*‐score was lower in HIV‐1–infected than HIV‐1–uninfected women: LS = −0.442 (1.141) versus 0.000 (0.985) g/cm^2^, *p* = 0.002; TH = −0.521 (1.125) versus 0.000 (0.985) g/cm^2^, *p* < 0.001; and FN = −0.471 (1.094) versus 0.000 (−0.985) g/cm^2^, *p* = 0.001, respectively (Table 1). Significant differences remained after controlling for age and BMI. The mean difference was −0.504 (95% CI, −0.783 to −0.226) at the LS, −0.613 (95% CI, −0.877 to −0.349) at the TH, and −0.541 (95% CI, –0.802 to −0.280) at the FN. It was *p* < 0.001 for all sites (Table 3).

**Table 2:** Proportion of Women with low BMD Based on Local and NHANES (2005–2008) Age‐Matched Reference Data

Study group by HIV‐1 status and contraception status	Local Ugandan reference data	NHANES non‐Hispanic White reference data	NHANES non‐Hispanic Black reference data
HIV‐1–uninfected nonhormonal users^a^	2.9% (2/69)	8.7% (6/69)	14.5% (10/69)
HIV‐1–infected nonhormonal users^b^	15.0% (33/220)	19.1% (42/220)	26.4% (58/220)
HIV‐1– infected previous DMPA users^c^	12.7% (7/55)	16.4% (9/55)	21.8% (12/55)
HIV‐1– infected current DMPA users^d^	20.3% (36/177)	23.7% (42/177)	28.8% (51/177)

Low BMD was defined as *Z*‐score ≤−2 at any of the three sites: lumbar spine, total hip, and femoral neck.

DMPA = depot medroxyprogesterone acetate; HIV‐1 = human immunodeficiency virus; IUD = intrauterine device; NHANES = National Health and Nutrition Examination Survey.

^a^ HIV‐1–uninfected women using IUD or condoms at baseline, and had not used DMPA or any other hormonal‐contraceptive method for more than three consecutive months in the last 2 years.

^b^ HIV‐1–infected women using IUD or condoms at base line, and had not used DMPA or any other hormonal‐contraceptive method for more than three consecutive months in the last 2 years.

^c^ HIV‐1–infected women not using DMPA for at least 6 months prior to enrollment but had ever used DMPA in the past regardless of the duration.

^d^ HIV‐1–infected women using DMPA at the time of enrollment for at least six consecutive months.

**Table 3:** Differences in Adjusted Mean BMD *Z*‐Scores at the Lumbar Spine, Total Hip, and Femoral Neck by HIV‐1 Status and Contraceptive Method

Comparison groups	Lumbar spine		Total hip		Femoral neck	
	BMD *Z*‐score mean difference g/cm^2^ (95% CI)^e^	*p* Value	BMD *Z*‐score mean difference g/cm^2^ (95% CI)^e^	*p* Value	BMD *Z*‐score mean difference g/cm^2^ (95% CI)^e^	*p* Value
HIV‐1–infected women^b^ ^,^ ^c^ ^,^ ^d^ vs. uninfected nonhormonal users^a^ (*N* = 521)	−0.504 (−0.783 to −0.226)	<0.001	−0.613 (−0.877 to −0.349)	<0.001	−0.541 (−0.802 to −0.280)	<0.001
HIV‐1–infected nonhormonal^b^ vs. uninfected nonhormonal users^a^ (*n* = 289)	−0.361 (−0.658 to −0.065)	0.017	−0.422 (−0.707 to −0.137)	0.004	−0.363 (−0.640 to −0.086)	0.010
HIV‐1–infected previous DMPA users^c^ vs. uninfected nonhormonal users^a^ (*n* = 124)	−0.419 (−0.830 to −0.007)	0.046	−0.611 (−0.969 to −0.253)	0.001	−0.611 (−0.987 to −0.234)	0.002
HIV‐1–infected previous DMPA users^c^ vs. infected nonhormonal users^b^ (*n* = 275)	−0.027 (−0.372 to 0.318)	0.878	−0.167 (−0.490 to 0.157)	0.312	−0.170 (−0.486 to 0.145)	0.288
HIV‐1–infected current DMPA users^d^ vs. uninfected hormonal users^b^ (*n* = 245)	−0.741 (−1.030 to −0.452)	<0.001	−0.873 (−1.144 to −0.603)	<0.001	−0.788 (−1.064 to −0.512)	<0.001
HIV‐1–infected current DMPA^d^ vs. infected nonhormonal users^a^ (*n* = 398)	−0.395 (−0.610 to –0.180)	<0.001	−0.485 (−0.693 to −0.277)	<0.001	−0.485 (−0.693 to −0.277)	<0.001

Codes below that correspond to the different study groups have been maintained from Table 2 for consistency.

DMPA = depot medroxyprogesterone acetate; HIV = human immunodeficiency virus; IUD = intrauterine device.

^a^ HIV‐1–uninfected women using IUD or condoms at base line, and had not used DMPA or any other hormonal‐contraceptive method for more than three consecutive months in the last 2 years.

^b^ HIV‐1–uninfected women using IUD or condoms at baseline, and had not used DMPA or any other hormonal‐contraceptive method for more than three consecutive months in the last 2 years.

^c^ HIV‐1–infected women not using DMPA for at least 6 months prior to enrollment but had ever used DMPA in the past regardless of the duration.

^d^ HIV‐1–infected women sing DMPA at the time of enrollment for at least six consecutive months.

^e^ Adjusted for age and BMI.

### BMD *Z*‐scores in untreated HIV‐1–infected nonhormonal users versus HIV‐1–uninfected women

HIV‐1–infected nonhormonal users had significantly lower mean BMD *Z*‐scores compared with the age‐matched HIV‐1–uninfected women; adjusted mean *Z*‐score difference at the LS was −0.361 (95% CI, −0.658 to −0.065), *p* = 0.017; at the TH it was −0.422 (95% CI, −0.707 to −0.137), *p* = 0.004; and at the FN it was −0.363 (95% CI, −0.640 to −0.086), *p* = 0.010 (Table 3). A significantly higher proportion of HIV‐1–infected nonhormonal users had low BMD (*Z*‐score <−2) compared with HIV‐1–uninfected women (*p* < 0.001; Table 3).

### BMD *Z*‐scores in untreated HIV‐1–infected previous and current DMPA users versus HIV‐1–uninfected women

A higher proportion of HIV‐1–infected previous and current DMPA users had low BMD compared with HIV‐1–uninfected women, 12.7% (7 of 55) and 20.3% (36 of 177) versus 2.9% (2 of 69), respectively (Table 2).

Compared with HIV‐1–uninfected women, HIV‐1–infected previous and current DMPA users had significantly lower mean BMD *Z*‐scores, the mean difference at the LS was −0.419 (95% CI, −0.830 to −0.007), *p* = 0.046; at the TH it was −0.611 (95% CI, −0.969 to −0.253), *p* = 0.001; and at the FN it was −0.611 (95% CI, −0.987 to −0.234), *p* = 0.002. Greater differences were observed when we compared HIV‐1–infected current DMPA users to HIV‐1–uninfected women; the mean difference at the LS was −0.741 (95% CI, −1.030 to −0.452), at the TH it was −0.873 (95% CI, −1.144 to −0.603), and at the FN it was −0.788 (95% CI, −1.064 to −0.512). It was *p* < 0.001 for all sites (Table 3). Similarly, HIV‐1–infected current DMPA users had the largest percentage difference in mean BMD between HIV‐1–infected and HIV‐1–noninfected groups (Table 4).

**Table 4:** Percentage Differences in Mean BMD Between HIV‐1–Infected and HIV‐1–Uninfected Controls

Body site	HIV‐1–infected nonhormonal users^a^	HIV‐1–infected previous DMPA users^b^	HIV‐1–infected current DMPA users^c^
Lumbar spine	−2.7%	−3.0%	−5.6%
Total hip	−3.3%	−4.9%	−7.5%
Femoral neck	−3.3%	−5.9%	−8.0%

DMPA = depot medroxyprogesterone acetate; HIV‐1 = human immunodeficiency virus; IUD = intrauterine device.

^a^ HIV‐1–uninfected women using IUD or condoms at base line, and had not used DMPA or any other hormonal contraceptive method for more than three consecutive months in the last 2 years.

^b^ HIV‐1–infected women not using DMPA for at least 6 months prior to enrollment but had ever used DMPA in the past regardless of the duration.

^c^ HIV‐1–infected women sing DMPA at the time of enrollment for at least six consecutive months.

### BMD Z‐scores in untreated HIV‐1–infected previous and current DMPA users versus HIV‐1–infected nonhormonal users

When we restricted the analysis to only HIV‐1–infected women, current DMPA users had a higher proportion of low BMD (*Z*‐score <2) at any of the three sites compared with either HIV‐1–infected previous DMPA users or nonhormonal users (Table 2).

After adjusting for age and BMI, there were no significant differences in mean BMD *Z*‐scores at any of the sites between untreated HIV‐1–infected DMPA previous users and HIV‐1–infected nonhormonal users, the adjusted mean difference at the LS was −0.027 (95% CI, −0.372 to 0.318), *p* = 0.878; at the TH it was −0.167 (95% CI, −0.490 to 0.157), *p* = 0.312; and the FN it was −0.170 (95% CI, −0.486 to 0.145), *p* = 0.288 (Table 3). However, significant differences were observed between HIV‐1–infected current DMPA and nonhormonal users at all sites, the mean difference at the LS was −0.395 (95% CI, −0.610 to −0.180), at the TH it was −0.485 (95% CI, −0.693 to −0.277); and at the FN it was −0.485 (95% CI, −0.693 to −0.277), after adjusting for age and BMI*, p* < 0.001 for all (Table 3).

### Comparison with NHANES (2005–2008) non‐Hispanic White and Black reference data

When we compared our data with the NHANES reference ranges rather than with the local non‐HIV‐1–infected Ugandan controls, a higher prevalence of low BMD was observed across all study groups regardless of DMPA or HIV‐1 status (Table 2). Using the NHANES Black reference database, the mean BMD *Z*‐scores in our control population were lower: LS = −1.156 (SD, 0.758), TH = −0.263 (SD, 0.783), and FN = −0.409 (SD, 0.764). Similar results were observed at the LS: LS = −0.843 (SD, 0.904) g/cm^2^ when the non‐Hispanic White data were used, but slightly higher values at the TH and FN: TH = 0.226 (SD, 0.921) g/cm^2^ and FN = 0.123 (SD, 0.963) g/cm^2^ (Table 5). Compared with US White women, mean BMD for Ugandan women was 11.3% to 13.6% lower at the LS, as well as at the TH (1.3%‐2.0% lower) and FN (2.3%‐6.5% lower), except for the 30‐ to 35‐year age category (1.0% and 1.4% higher at the TH and FN, respectively). Greater differences were observed compared with US Black women; mean BMD for Black Ugandan women was lower at all sites for all age categories; 19.1% to 19.4% at the LS, 4.7% to 9.2% at the TH, and 5.5% to 14.6% at the FN.

**Table 5:** BMD *Z*‐Scores and Percent Differences in Mean BMD Between the Local Ugandan Control Group and NHANES (2005–2008) Age‐Matched Reference Data

Body site	NHANES non‐Hispanic White reference data	NHANES non‐Hispanic Black reference data
Mean BMD *Z*‐score (g/cm^2^)		
Lumbar spine	−0.843	−1.156
Total hip	0.226	−0.263
Femoral neck	0.123	−0.409
Percent differences in mean BMD (g/cm^2^)		
Lumbar spine		
Below 20 y	−11.3	−19.4
20–29 y	−13.6	−19.3
30–35 y	−12.2	−19.1
Total hip		
Below 20 y	−2.0	−9.2
20–29 y	−1.3	−8.0
30–35 y	1.0	−4.7
Femoral neck	
Below 20 y	−6.5	−14.6
20–29 y	−2.3	−11.3
30–35 y	1.4	−5.5

NHANES = National Health and Nutrition Examination Survey.

## Discussion

In this cross‐sectional analysis of baseline data from an ongoing longitudinal study, we observed a significantly higher prevalence of low BMD and mean differences in BMD *Z*‐scores among HIV‐1–infected ART‐naïve Ugandan women compared with HIV‐1–uninfected women independent of age and BMI.^(^ ^29^, ^30^ ^)^ Current DMPA users had lower BMD at all three sites than previous DMPA users or nonhormonal‐contraceptive users, suggesting BMD recovery after DMPA discontinuation.

The prevalence of low BMD in this population of HIV‐1–infected ART‐naïve women is generally higher than that reported in previous studies using the same definition of low BMD (*Z*‐scores ≤−2 at any of the three sites). In a study conducted by Brown and colleagues among 331 HIV‐1‐infected individuals,^(^ ^31^ ^)^ low BMD was observed in 33 subjects (10%) prior to ART initiation. Similarly, in the multicenter START (Strategic Timing of Antiretroviral Treatment) BMD substudy conducted at 33 sites in 11 countries, the proportion of participants with low BMD pre‐ART initiation was 11.3%.^(^ ^32^ ^)^ Of note, the above studies enrolled primarily older men with small numbers of HIV‐1‐infected women,^(^ ^31^, ^32^ ^)^ and although in the START BMD substudy *Z*‐scores were standardized relative to age‐, sex‐, and race/ethnicity‐ (Black/White/Hispanic) matched reference populations, White reference populations were used for all other races/ethnicities (about 60% of study population).^(^ ^32^ ^)^ Our finding of significant differences in BMD *Z*‐scores among HIV‐1–infected nonhormonal users compared with the uninfected internal comparison group show the independent effect of HIV‐1 on BMD.

The above findings are consistent with the majority of published studies that have reported HIV‐1 to be an independent risk factor for low BMD^(^ ^33^, ^34^, ^35^, ^36^, ^37^ ^)^ and can be explained by the direct effect of the HIV‐1 virus, as well as chronic immune activation. In untreated HIV‐1, through direct viral effects and inflammatory effects, bone resorption and bone formation are uncoupled. With chronic HIV‐1 infection, there is persistent stimulation of *T* cells by HIV‐1 viral proteins and an increase in the synthesis of proinflammatory cytokines, such as IL‐1, IL‐6, and TNF‐α.^(^ ^38^, ^39^, ^40^ ^)^ Cytokines suppress osteoclast apoptosis and stimulate osteoclast activity,^(^ ^41^, ^42^, ^43^, ^44^ ^)^ thereby increasing bone resorption.^(^ ^45^, ^46^, ^47^ ^)^ Similarly, high concentrations of HIV‐1 RNA have been associated with elevated levels of RANKL, an osteoblast‐secreted cytokine that promotes osteoclast formation.^(^ ^48^ ^)^ However, in our study population, no association was observed between low BMD and viral load or CD4 cell count. A similar cross‐sectional analysis by Hamill and colleagues assessing BMD in HIV‐1‐infected South African women at different disease stages (as measured by CD4 cell count) versus HIV‐uninfected women did not show any differences in BMD at any of the sites.^(^ ^49^ ^)^ The lack of difference between HIV‐1–infected and HIV‐1–uninfected women in this study is likely caused by the protective effective of high BMI in this population with a median BMI of 26.1 kg/m^2^ (IQR, 22.4‐31 kg/m^2^).

We further observed significantly lower mean BMD *Z*‐scores among HIV‐1–infected current DMPA users compared with both HIV‐1–infected nonhormonal users and HIV‐1–infected previous DMPA users. Our finding of a strong negative effect of current DMPA use on BMD is consistent with previous studies conducted among the general population, in whom BMD losses of up to 7.5% of BMD after 2 or more years of use were recorded with the greatest loss being during the first 1 to 2 years.^(^ ^50^, ^51^ ^)^ Because of the hypoestrogenic effects of DMPA, current DMPA users have been shown to have lower mean BMD than nonusers, and these effects are more pronounced in younger women.^(^ ^51^, ^52^ ^)^ Post hoc exploratory analysis showed that untreated HIV‐1–infected current DMPA users had lower BMD compared with untreated nonhormonal users regardless of disease status or CD4 cell count (data not shown). These results imply that DMPA has a stronger negative effect on BMD than HIV‐1 itself, and the severity of HIV as measured by a decline in CD4 cell count may play a smaller role in the process of BMD loss.

Comparing the differences in mean BMD values among the four study groups, the greatest difference was between HIV‐1–infected current DMPA users and the nonhormonal control group (5.6%–8%). Our search of published articles did not yield any previously published data on BMD among HIV‐1–infected DMPA users. On the other hand, we found no differences in BMD between HIV‐1–infected DMPA previous users and HIV‐1–infected nonhormonal users. This finding provides evidence of BMD recovery following the cessation of DMPA use consistent with results from a number of studies conducted among the general population, which have reported that the adverse effect of DMPA on bone may be temporary and that bone mass may recover after discontinuation of the contraceptive, ultimately reaching levels close to those seen in women not on DMPA.^(^ ^21^, ^22^ ^)^

To our knowledge, this is one of the few studies to systematically evaluate BMD among HIV‐1–infected women globally and to further estimate the independent effect of DMPA on BMD. We provide the first evidence on the combined effect of DMPA and HIV‐1 on bone mass. One of the main strengths of our study is the use of an internal HIV‐1–uninfected age‐matched, Ugandan reference population as opposed to the NHANES or Hologic reference ranges based on data from US Black and White populations, which do not represent the BMD of sub‐Saharan Black women.^(^ ^53^ ^)^ Indeed, analysis using NHANES reference ranges for White non‐Hispanic women gives a higher prevalence of low BMD among both the HIV‐infected ART‐naïve women and the uninfected comparison group of 20.6% (93 of 452) and 8.7% (6 of 69), respectively, at the same Z‐score cut‐off (Table 2). Compared with African American women, the proportion of low BMD among HIV‐1–infected and HIV‐1–uninfected women is even higher: 26.8% (121 of 452) and 14.5% (10 of 69), respectively. Compared with non‐Hispanic White and Black women, the Ugandan local reference population had generally lower mean BMD at all sites for the majority of age categories consistent with data from Zimbabwe.^(^ ^53^ ^)^ In addition, our study population comprised untreated HIV‐1–infected women; thus, the findings exclude the possible confounding effect of ART treatment. Finally, our sample size of 521 women including 452 HIV‐1–infected women provides high power to detect significant differences for our current analyses.

Our study has some limitations. Our cross‐sectional data do not allow us to evaluate causal relationships between HIV‐1 infection and DMPA use on BMD. In addition, the lack of assessment of PTH, 25 hydroxyvitamin D, bone‐turnover biomarkers, or cytokine levels precludes us from drawing more detailed conclusions about the underlying mechanisms of the observed associations. We plan to examine some of these factors in further analyses. In addition, our study was comprised of only Ugandan women, which may limit the generalizability of our findings. However, the results are more likely to generalize to other populations in sub‐Saharan Africa than studies done on other continents.

In summary, adult Ugandan HIV‐1–infected ART‐naïve women showed low BMD compared with their HIV‐1–uninfected counterparts. Significantly lower BMD was seen among HIV‐1–infected women currently using DMPA. As HIV‐1 is a chronic disease, our findings provide insight into the prevalence of low BMD in untreated HIV‐1–infected women on DMPA and nonhormonal contraception in a resource‐limited setting before long‐term exposure to ART. Given that lower BMD may predispose HIV‐infected subjects to increased morbidity and further bone loss with ART exposure, the planned longitudinal analysis of this same cohort provides a unique opportunity to understand the additional impacts of ART on bone mass among HIV‐1–infected women particularly on DMPA.

## Disclosures

None of the authors has financial, consultant, institutional, or other relationships that might lead to a bias or conflict of interest.

## Author Contributions

**Flavia Matovu:** Conceptualization; funding acquisition; investigation; methodology; project administration; supervision; validation; visualization; writing‐original draft. **Noah Kiwanuka:** Formal analysis; supervision; writing‐review and editing. **Martin Nabwana:** Data curation; formal analysis; writing‐review and editing. **Esther Isingel:** Methodology; project administration; writing‐review and editing. **Delia Scholes:** Conceptualization; funding acquisition; methodology; visualization; writing‐review and editing. **Monica Nolan:** Resources; supervision; writing‐review and editing. **Philippa Musoke:** Resources; supervision; writing‐review and editing. **Mary Fowler:** Conceptualization; funding acquisition; resources; supervision; visualization; writing‐review and editing. **John Pettifor:** Conceptualization; investigation; methodology; supervision; visualization; writing‐review and editing. **Todd Brown:** Conceptualization; funding acquisition; investigation; methodology; supervision; visualization; writing‐review and editing. **Mags Beksinska:** Conceptualization; investigation; methodology; supervision; visualization; writing‐review and editing.

### Peer Review

The peer review history for this article is available at https://publons.com/publon/10.1002/jbm4.10446.
